# Readiness for Practice and Associated Factors Among Baccalaureate Nursing Students in Mongolia: A Mixed Methods Study

**DOI:** 10.3390/nursrep15110409

**Published:** 2025-11-20

**Authors:** Dulamsuren Damiran, Taewha Lee, Sue Kim, Wonhee Lee, Jiyeon Choi, Chang Gi Park

**Affiliations:** 1 School of Nursing, International University of Ulaanbaatar, Ulaanbaatar 210644, Mongolia; dulamsuren.da@ulaanbaatar.edu.mn (D.D.); wonhee.l@ulaanbaatar.edu.mn (W.L.); 2 Brain Korea 21 Four Project, Mo-Im Kim Institute, College of Nursing, Yonsei University, Seoul 03722, Republic of Korea; 3 Mo-Im Kim Institute, College of Nursing, Yonsei University, Seoul 03722, Republic of Korea; suekim@yuhs.ac (S.K.); jychoi610@yuhs.ac (J.C.); 4 Department of Population Health Nursing Science, College of Nursing, University of Illinois Chicago; Chicago, IL 60612, USA; parkcg@uic.edu

**Keywords:** nursing students, clinical competence, education, nursing, baccalaureate, preceptorship, mixed methods research

## Abstract

**Background/Objectives:** Readiness for practice is an essential outcome of nursing education, yet the factors influencing it among baccalaureate nursing students in Mongolia remain underexplored. This study aimed to provide a holistic understanding of factors influencing readiness for practice among baccalaureate nursing students in Mongolia, employing both quantitative and qualitative approaches. **Methods:** A convergent mixed-methods design was used. The study included 150 final-year baccalaureate nursing students from 14 Mongolian universities. Quantitative data were collected via survey and analyzed using multiple regression analyses in SPSS 26.0. Concurrently, qualitative data were obtained through focus group interviews with 25 participants (nurses and faculty) and analyzed using content analysis. **Results:** Quantitative analyses revealed that the clinical learning environment, clinical competence, and critical thinking significantly influenced readiness for practice, explaining 40% of the variance. Qualitative findings—derived from nurses’ and faculty’s perspectives and findings—provided deeper insights: “*maturity*” was defined as students’ coping ability and adaptability; “*competence*” encompassed clinical, ethical, cultural, and communication skills; and “*professional values*” reflected passion, motivation, and readiness to engage in practice. These findings highlighted the essential interplay between personal, educational, and contextual factors in shaping readiness. **Conclusions:** Findings suggest strategies to enhance nursing students’ readiness, including fostering supportive clinical learning environments, structured mentorship, and integrating ethical and cultural training into curricula. These insights offer actionable recommendations for nursing schools and clinical institutions to strengthen collaboration, support professional development, and prepare competent, adaptable, and ethically grounded nursing graduates in Mongolia.

## 1. Introduction

Advances in technology, emerging health challenges such as pandemics, and evolving diseases continually shape the global healthcare landscape. The intricate nature of the ever-changing healthcare system, coupled with economic constraints, the escalating medical needs of an aging population, and the anticipated nursing shortage, underscores the importance of ensuring that new nurses are adequately prepared for professional practice [1]. However, the retention of new graduate nurses remains a global challenge, with many facing difficulties during the transition to practice, leading to a significant number leaving their positions [2,3]. Disturbingly, 30–60% of graduate nurses change jobs or exit the nursing profession within their initial year of practice, with challenges in role transition being a major factor influencing this decision [1,3]. Recognizing the pivotal role of nursing educators in facilitating a successful transition, researchers have focused on the transition from nursing student to graduate nurse for decades, emphasizing the crucial role played by educators in this process [4]. Additionally, Benner’s seminal theory in 1984 affirmed that professional growth continues post-graduation, emphasizing the educator’s role in preparing students for this ongoing development [5].

This concern about nursing students’ readiness extends to a global exploration of the concept, which involves the competence, information, abilities, and judgment required for role performance [1]. However, it remains uncertain whether students are adequately prepared to assume the role and responsibilities of a registered nurse [2]. Consequently, the factors contributing to the perceived preparedness of new graduates and how education and practice can better prepare them continue to be integral to ongoing scholarly discourse [6]. Previous research has identified various personal characteristics influencing nursing students’ readiness for practice, including financial support, prior working experience, age, and school type [3,7,8,9]. Studies have also demonstrated the impact of professional competence on readiness for practice [2,10]. Conversely, the lack of competence and ability to perform basic clinical procedures has been identified as a hindrance to graduate students’ readiness [11]. Moreover, the clinical learning environment, characterized by its nature, the significance attributed to nurses’ work, the incorporation of real nursing culture, and an awareness of the nursing role, has emerged as a significant predictor of student readiness for practice [12].

Nursing education in Mongolia is provided through three- and four-year programs implemented by universities and medical colleges under the oversight of the Ministry of Education and Science and the Ministry of Health. Nonetheless, the system continues to confront significant challenges, such as limited access to clinical placements, a shortage of simulation and laboratory infrastructure, and disparities in curricular quality across educational institutions.

The Mongolian healthcare system continues to be primarily centered on hospital-based services, with nurses assuming essential responsibilities in both primary and tertiary levels of care. However, nurses often face a lack of mentorship, substantial workload burdens, and restricted opportunities for ongoing education and career advancement. Such contextual constraints may adversely affect the preparedness and self-assurance of new graduate nurses entering professional practice.

A review of national literature and reports indicated that only a limited number of studies have explored nurses’ preparedness or readiness for clinical practice. A search of the Mongolian Health Science Database and national academic repositories covering the years 2010–2023 identified merely two studies that indirectly addressed nursing competence and clinical performance, with none specifically investigating nursing students’ readiness for practice. Furthermore, data from the Ministry of Health [13] revealed that newly graduated nurses often require prolonged adjustment periods in their initial employment, suggesting potential deficiencies in practice readiness [13,14].

Moreover, a previous doctoral dissertation identified nursing students’ preparedness as a predictive factor influencing the quality of nursing care in Mongolia [14].

However, the study did not examine additional variables associated with readiness for practice. Consequently, comprehensive exploratory research is needed to investigate readiness for practice from multiple perspectives within the Mongolian context. These findings underscore the paucity of existing research and emphasize the necessity of gaining a more comprehensive understanding of the factors influencing readiness for practice within Mongolian nursing students.

Therefore, this study seeks to address this gap by examining readiness for practice among baccalaureate nursing students in Mongolia through both student self-assessments and the perspectives of nurses and faculty. The findings are expected to inform evidence-based improvements in undergraduate nursing curricula and orientation programs for new nurses.

Research questions:

1. What is the level of readiness for practice among baccalaureate nursing students in Mongolia?
2. What factors are associated with nursing students’ readiness for practice?
3. How do nurses and faculty members perceive the readiness of nursing students for clinical practice?
4. What themes emerge from the qualitative exploration of readiness for practice within the Mongolian context?

## 2. Methods

### 2.1. Study Design

**Figure 1:**
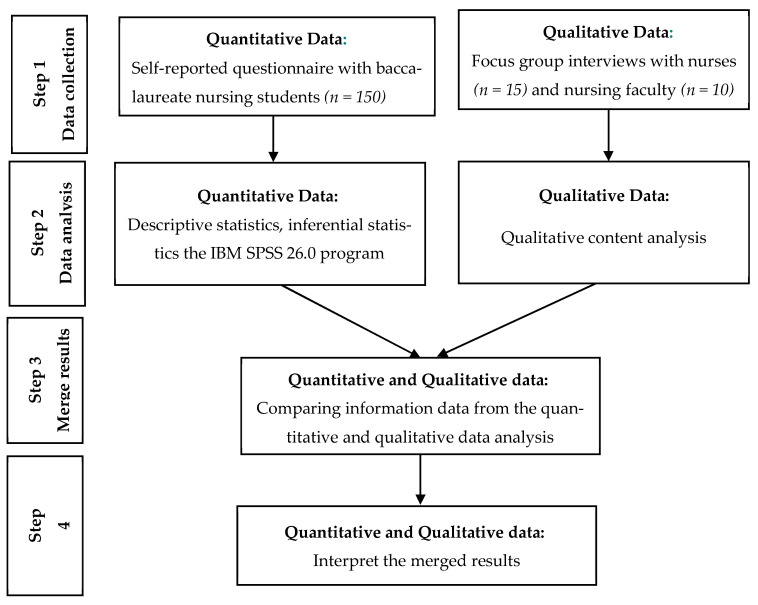
Flowchart of the convergent mixed methods design of this study [17,18].

A convergent mixed-methods approach [15] was adopted to investigate the determinants of readiness for practice among Mongolian baccalaureate nursing students (Figure 1). Quantitative and qualitative data were collected concurrently but from different participant groups to ensure complementary perspectives. The quantitative strand included survey responses from 150 final-year nursing students regarding their readiness and influencing factors, while the qualitative strand comprised focus group discussions with 15 nurses and 10 faculty members to provide contextual insights. Each dataset was analyzed separately using appropriate statistical and content analysis methods, after which integration was achieved through a joint display technique to confirm, extend, or explain the findings and to yield a holistic interpretation of readiness for practice [15,16]. This design allowed for the meaningful integration of measurable outcomes with experiential perspectives. The study followed the Mixed Methods Article Reporting Standards (MMARS) to maintain methodological transparency and rigor [16].

### 2.2. Measures

Readiness for practice. Readiness for practice was measured by the Casey-Fink Preparation for Practice Survey (CFRPS), including 20 items developed by Casey et al. [1]. The items consisted of four domains: clinical problem-solving, learning techniques, professional identity, and trials and tribulations. The response rates ranged from 1 (strongly disagree) to 4 (strongly agree) based on a Likert scale. A total score was calculated as the average of item scores. Cronbach’s alpha ranged from 0.50 to 0.80 for the four subscales [19] and 0.71 for this study.

Clinical learning environment. The clinical learning environment was measured by the Clinical Learning Environment Supervision scale, using 27 items developed by Saarikoski and Leino-Kilpi (2002) [20]. This instrument includes five sub-dimensions: ward atmosphere, leadership style of the ward manager, premises of nursing care on the ward, premises of learning on the ward, and supervisory relationship. Responses ranged from 1 (fully disagree) to 5 (fully agree). The score of each sub-dimension is the average of item scores in that sub-dimension. Cronbach’s alpha ranged from 0.73 to 0.94 for the five sub-dimensions [19] and 0.93 for this study.

Clinical nursing competence. Clinical nursing competence was measured using 22 items developed by Lee-Hsieh et al. (2003) [21]. This instrument includes four dimensions: caring, communication and coordination, management/teaching, and professional self-growth. Responses ranged from 1 (never) to 5 (always). The total score possible for all 22 items ranged from 22 to 100. Cronbach’s alpha was 0.93 for all items [19] and 0.94 for this study.

Critical thinking. Critical thinking was measured by the Critical Thinking Disposition scale of Shin et al. (2015) [22], including 20 items. Critical thinking disposition was composed of intellectual eagerness/sound skepticism (7 items), intellectual honesty (6 items), prudence (4 items), and objectivity (3 items). The instrument ranged from 20 to 100 points. The Cronbach’s alpha for the previous study was 0.73, and for the current study it was 0.85.

Professional value. Professional value was measured by the Nurses Professional Values Scale-3 (NPVS-3), including 28 items developed by Weis and Schank (2017) [23]. The items consisted of three domains: caring (10 items), activism (10 items), and professionalism (8 items). Response rates ranged from 1 (not important) to 5 (most important). The total score ranged from 28 to 140. Cronbach’s alpha was 0.94 for the instrument [19] and 0.91 for this study.

Readiness for practice-related sample characteristics. Sample characteristics were measured with 8 items. Maturity included age, gender, marital status, pursuing a second degree, prior working experiences, learning performance (GPA), and cultural background, including motivation to choose the nursing profession and financial support, all derived from the literature [3,6,7,9,24,25].

### 2.3. Ethical Considerations

Ethical approval for the study was granted by the Institutional Review Board of the Mongolian National University of Medical Sciences (IRB No. Y-2022/3–09).

In the quantitative phase, participants received written information outlining the study’s purpose, procedures, potential risks and benefits, confidentiality measures, and the voluntary nature of participation. The first page of the online survey contained a detailed consent statement, and participants were required to provide electronic consent before accessing the questionnaire. They were assured that their responses would remain anonymous and that they could discontinue participation at any point without consequence.

For the qualitative phase, participants were provided with verbal and written explanations of the study’s purpose, the audio-recording procedures, confidentiality measures, and their right to refuse to answer any question or withdraw at any time without consequence. Written informed consent was obtained from all participants prior to participation and before the commencement of audio recording. Identifying information was removed during transcription, and all data were securely stored on password-protected devices accessible only to the research team.

### 2.4. Data Collection

Data collection consisted of completing online self-report questionnaires and attending focus group interviews. The questionnaire was collected through an online survey targeting students who understood the purpose of the study and agreed to voluntary participation. All participants received contact information in case they had questions about the survey questionnaire. It took 20–25 min to complete the questionnaire. The standardized instruments employed in this study—the Casey-Fink Readiness for Practice Survey (CFRPS), the Nurse Professional Values Scale-3 (NPVS-3), the Clinical Learning Environment Supervision Scale, the Clinical Nursing Competence Scale, and the Critical Thinking Disposition Scale—were originally developed in English. To ensure both linguistic and conceptual equivalence for use in the Mongolian context, a double forward translation procedure was conducted by two bilingual nursing experts proficient in English and Mongolian. The resulting translations were compared, reconciled, and subsequently reviewed by a panel of nursing faculty to evaluate content relevance and cultural appropriateness.

Although a full back-translation and comprehensive psychometric validation were not conducted at this stage, content validity and face validity were evaluated by a panel of five Mongolian nursing education experts. Minor revisions were made to improve clarity and contextual appropriateness. The Mongolian versions demonstrated acceptable internal consistency in this study, with Cronbach’s α values ranging from 0.81 to 0.89 across instruments, indicating satisfactory reliability for exploratory research purposes.

### 2.5. Sample and Data Collection Section

A total of 150 final-year baccalaureate nursing students were recruited through convenience sampling. Eligible participants were (1) enrolled in the final semester of a four-year baccalaureate nursing program, (2) had completed all required clinical practicum courses, and (3) voluntarily agreed to participate in the study.

The required sample size for the quantitative phase was calculated using G*Power 3.1 for multiple linear regression. The analysis was based on a medium effect size (f^2^ = 0.10), α = 0.05, and power (1 – β) = 0.80. The effect size (f^2^ = 0.10) was determined based on previous studies examining predictors of readiness for practice and clinical competence among nursing students [2,8,10], which typically reported small-to-medium effect sizes in similar models.

The initial sample size estimation considered up to 26 potential predictors derived from variables reported in prior research related to readiness for practice (e.g., personal, educational, and environmental factors). However, only the most theoretically relevant predictors—clinical learning environment, clinical competence, critical thinking, and professional values—were included in the final regression analysis. The power analysis indicated a minimum of 135 participants, and 150 were recruited to account for potential nonresponse or incomplete data.

For the qualitative component, purposive sampling was used to select 25 participants, including 15 clinical nurses and 10 nursing faculty members, who had direct experience supervising or teaching final-year nursing students. Inclusion criteria for both groups were (1) having at least one year of experience in clinical education or student supervision and (2) willingness to share their perspectives. The sample size was guided by the principle of data saturation, which was reached when no new themes emerged during analysis.

The analysis systematically compared and contrasted the perspectives of nurses and faculty members to identify both commonalities and differences regarding nursing students’ readiness for practice, ensuring that diverse viewpoints were captured and meaningfully integrated.

For focus group interview, the interviews were driven by five questions: (1) Please share your clinical experience ready to work as a nurse. (2) In your experience, how have you observed the transition from student to nurse, and what challenges have you noticed? (3) In your interactions with nursing students, have you observed any specific factors that influence their readiness for practice? (4) What skills and behaviors do you think are reflected in well-prepared nurses? and (5) What are your recommendations for feeling more prepared? The average length of each focus group interview was approximately 90 min (range was 75–105 min). During the interview, participants could answer freely but ensured that the content did not deviate from the research topic and interfere with the interview flow. The FGIs were audiotape recorded upon agreement of participants. Audiotapes were transcribed verbatim by the researcher who had been trained in qualitative transcription procedures. Data were collected from December 2022 to August 2023.

### 2.6. Data Analysis

The quantitative phase data analysis was performed using IBM SPSS 26.0. Descriptive statistics, including means, standard deviations (SD), and proportions, were calculated to study the general characteristics of the participants. Sample normality was verified by calculating the sample mean and SD, and Pearson’s correlation coefficient was used to identify relationships among variables. Differences in measurement variables according to general characteristics were analyzed using *t*-tests, one-way ANOVA, and post hoc tests. The internal consistency of instruments was analyzed using Cronbach’s α, and factors influencing readiness for practice were examined using multiple regression analysis [2,8,10].

In the qualitative phase, the audio-recorded interviews (undertaken in the Mongolian language) were transcribed verbatim by the researcher. Participants’ names were replaced by number codes to ensure anonymity. Participants were shown the transcripts and invited to comment, but none provided feedback. Data were analyzed using a qualitative content approach with an inductive analytic method [17]. Transcript data were coded, condensing the text while retaining its core meaning. After independent coding by the researcher, coding was verified to identify commonalities and differences. During categorization, coded data with similar meanings were combined under themes representing perceptions of nursing students’ readiness for practice. Selected quotations were translated into English through double forward translation.

For the integration phase, results from the quantitative and qualitative analyses were merged. Meta-inferences were drawn on three constructs to determine whether the data confirmed, expanded, or were in discordance with one another. Confirmation indicates that one analysis supports the results of the other; expansion indicates divergence, providing additional insights by describing complementary aspects of the construct; discordance indicates disagreement between quantitative and qualitative results [26]. Joint display analysis was used to integrate the two data types to achieve a comprehensive understanding [26].

## 3. Results

### 3.1. Quantitative Results

#### 3.1.1. Participant Demographics and Academic Characteristics

The mean age of the participants was 24.15 (SD = 4.56) years old, ranging from 19 to 42 years old. Most of the participants were female (97.3%), married (57.6%), and studying at public universities (52.7%) in the capital area (65.3%). Only 5.3% of them were pursuing a second degree, and 13.3% of participants were previously employed in healthcare institutions before enrolling in nursing school. Regarding the major reasons for choosing the nursing profession, academic interest and aptitude (56.6%) was the highest, followed by suggestion of parents (22.0%), and the mean GPA among the student participants was 3.12 (SD = 0.32) on a 4-point scale, indicating generally good academic performance (Table 1).

#### 3.1.2. Summary of Factors Associated with Readiness for Practice

Table 2 presents the descriptive statistics of readiness for practice and its associated factors among final-year nursing students. The mean score for readiness for practice was 57.8 ± 5.8 (possible range: 20–80), corresponding to 2.89 ± 0.29 on a 4-point scale, which indicates a *moderate-to-high level* of perceived readiness. Among the associated factors, the clinical learning environment had a mean score of 136.3 ± 20.7 (4.01 ± 0.61 on a 5-point scale), suggesting that students generally perceived their clinical learning settings positively. The clinical nursing competence mean score was 174.2 ± 21.9 (3.87 ± 0.60), reflecting a high level of self-perceived competence. The critical thinking mean score was 95.9 ± 14.7 (3.55 ± 0.60), showing a moderate level of critical thinking ability. Meanwhile, the professional value score was 106.8 ± 12.3 (4.11 ± 0.55), representing a strong endorsement of nursing professional values. Overall, the findings demonstrate that nursing students in this study reported moderate to high readiness for practice, supported by positive perceptions of their learning environments, solid clinical competence, and strong professional values.

#### 3.1.3. Relationship Analysis Between Readiness for Practice and Influencing Factors

Table 3 presents the correlations between readiness for practice and its associated factors. Pearson’s correlation analysis was conducted using continuous sample characteristics (age, GPA, and enrollment score) and the main study variables. The results indicated that readiness for practice was positively correlated with the clinical learning environment (r = 0.42, *p* < 0.001), clinical nursing competence (r = 0.48, *p* < 0.001), critical thinking (r = 0.45, *p* < 0.001), and professional value (r = 0.19, *p* < 0.05). No statistically significant correlations were found between readiness for practice and the continuous demographic variables (age, GPA, and enrollment score).

### 3.2. Factors Affecting Nursing Students’ Readiness for Practice

Table 4 presents the multiple linear regression analysis of factors affecting nursing students’ readiness for practice. Variables included in the model were selected based on theoretical frameworks and previous research, highlighting clinical learning environment, clinical competence, critical thinking, and professional values as key predictors. Control variables (age, gender, marital status, employment, school type/location, and financial support) were included to account for potential confounding effects. The results showed that clinical learning environment (β = 0.24, *p* = 0.001), clinical competence (β = 0.17, *p* = 0.005), and critical thinking (β = 0.28, *p* < 0.001) were significant positive predictors, explaining 40% of the variance in readiness for practice (Adjusted R^2^ = 0.409). Professional value and all demographic/control variables were not statistically significant predictors.

### 3.3. Qualitative Results

The qualitative analysis of FGIs with nurses and nurse faculty identified five major categories and 12 subcategories representing nursing students’ readiness for practice in Mongolia (Table 5). Maturity was highlighted by both groups as critical to readiness. Nurses emphasized students’ ability to cope with challenging clinical experiences and adapt to the workplace, noting, “Students who stay calm and think critically under pressure handle patients more safely.” Faculty highlighted students’ genuine desire to learn, stating, “Motivated students actively seek feedback and demonstrate professional growth.” Competence encompassed essential clinical skills, ethical and cultural competence, and communication skills. Nurses stressed the importance of communication in navigating complex clinical environments, while faculty emphasized confidence gained from demonstrating clinical proficiency. Communication and theory-practice integration were consistently noted. Nurses valued students who could apply classroom learning to patient care and adapt to dynamic situations, whereas faculty highlighted adaptability and the ability to integrate theory with practice effectively. Professionalism was a central theme across both groups, reflecting passion for nursing and understanding of the profession’s societal value. Nurses noted, “Passionate students naturally engage more in patient care,” and faculty added, “Understanding the role of nursing in society shows professional maturity.” These categories and subcategories were derived through content analysis, coding meaningful statements from transcripts, and grouping them into conceptual categories to reflect shared perceptions of readiness for practice.

### 3.4. Integrations

The joint display (Table 6) demonstrates that the clinical learning environment, clinical competence, and critical thinking are central factors influencing nursing students’ readiness for practice. Quantitative results indicated that a positive clinical learning environment directly enhanced readiness by improving both competence and critical thinking. Insights from the focus groups further elaborated on these findings, highlighting the importance of supportive clinical settings, preceptor guidance, and opportunities for hands-on practice. Additionally, integrating ethical and cultural competence into clinical experiences was identified as essential for preparing students to provide respectful care across diverse cultural and religious contexts. These findings suggest that ethical and cultural training should be incorporated into Mongolian nursing curricula to strengthen students’ knowledge, skills, and professional behavior.

The quantitative analysis indicated significant effects of clinical competence (β = 0.17, *p* = 0.005) and critical thinking (β = 0.28, *p* < 0.001), which were supported and enriched by qualitative data. Focus group insights highlighted additional dimensions, including ethical, cultural, and communication considerations, as well as the integration of theoretical knowledge into clinical practice.

An inconsistency emerged concerning professional values. Quantitative results suggested that professional values were not a significant predictor of readiness (β = −0.04, *p* = 0.537), whereas qualitative data emphasized their central role. Participants characterized professional values as including enthusiasm for nursing, appreciation of the profession’s significance, and the development of maturity, such as coping, adaptability, and a genuine motivation to learn. This inconsistency may reflect the distinction between espoused values (self-reported attitudes) and enacted values (observable behaviors in clinical settings). Moreover, cultural and contextual factors may limit the NPVS-3’s ability to fully capture professional values in the Mongolian context.

Although quantitative analysis indicated that professional values were not a significant predictor of nursing students’ readiness for practice (β = −0.04, *p* = 0.537), qualitative findings underscored their critical importance. Participants highlighted the significance of passion for nursing, appreciation of the profession’s value, and the development of maturity, including coping skills, adaptability, and a genuine commitment to learning. This discrepancy suggests that quantitative measures may not fully capture professional values, yet they are regarded as essential by both nurses and faculty for fostering readiness for practice. These findings demonstrate the value of mixed-method approaches in uncovering nuanced factors that may be overlooked by quantitative analysis alone.

## 4. Discussion

This study aimed to examine nursing students’ readiness for practice in Mongolia, identify key factors influencing readiness, and explore how nurses and nursing faculty perceive this readiness. The results indicated an average readiness score of 2.89 ± 0.29 on a 4-point scale, reflecting moderate readiness. This score is lower than those reported in the United States (3.40 ± 0.39) and the United Arab Emirates (3.05 ± 0.72) but comparable to findings from Korea (2.86 ± 0.31) [1,25,27]. Approximately 60% of senior nursing students reported feeling prepared for professional practice, consistent with findings from the United Arab Emirates and Turkey [2,27], but lower than readiness rates reported among Australian students [10]. These results suggest that cultural and contextual factors in Mongolia may influence students’ self-assessed preparedness for professional nursing roles.

The integration of quantitative and qualitative findings identified the clinical learning environment, clinical competence, and critical thinking as primary determinants of nursing students’ readiness for practice. Quantitative analysis revealed significant effects for the clinical learning environment (β = 0.24, *p* = 0.001), clinical competence (β = 0.17, *p* = 0.005), and critical thinking (β = 0.28, *p* < 0.001). Qualitative data further elaborated these results, highlighting the importance of supportive clinical placements, preceptor guidance, opportunities for hands-on practice, and the integration of ethical and cultural competence. These findings are particularly relevant in the Mongolian context, where clinical placements differ in structure, mentorship is inconsistent, and professional recognition for nurses may be limited. Such contextual factors likely affect students’ exposure to complex clinical situations, the development of critical thinking skills, and overall readiness for professional practice.

A notable mixed-methods finding was the discrepancy regarding professional values. While quantitative analysis indicated that professional values were not a significant predictor of readiness (β = −0.04, *p* = 0.537), qualitative data highlighted their critical role. Participants characterized professional values as including passion for nursing, appreciation of the profession’s significance, and maturity, encompassing coping skills, adaptability, and intrinsic motivation. This divergence may reflect the distinction between espoused values (students’ self-reported attitudes on the NPVS-3) and values-in-action (observable behaviors in clinical practice). In the Mongolian context, students may understand professional values conceptually but have limited opportunities to demonstrate them due to structural or cultural constraints within clinical placements, hierarchical norms in nursing practice, and limited mentorship. Furthermore, the NPVS-3 may not fully capture local interpretations of professionalism, underscoring the importance of qualitative approaches in revealing culturally contextualized perspectives.

The findings also highlight the significance of maturity and adaptability in nursing students’ readiness for practice. Focus group discussions consistently emphasized the importance of coping with challenging clinical experiences, adjusting to diverse workplace environments, and demonstrating intrinsic motivation. Together with clinical competence and critical thinking, these factors reflect the complex interaction of personal, educational, and contextual influences shaping readiness for practice in Mongolia.

Overall, the integration of quantitative and qualitative findings offers a comprehensive understanding of nursing students’ readiness for practice. While quantitative measures assess observable competencies, qualitative data provide insight into underlying attitudes, values, and contextual factors that are critical for professional development. Implementing structured mentorship, culturally relevant ethical training, and opportunities for integrating theory and practice within Mongolian nursing curricula may enhance students’ readiness and address gaps identified in this study. Beyond educational outcomes, improving readiness for practice also carries important economic and policy implications. A more practice-ready nursing workforce can reduce early-career turnover, lower orientation and retraining costs, and enhance workforce stability in hospitals and community health settings. A nursing workforce that is better prepared for practice can help reduce early-career turnover, decrease orientation and retraining costs, and promote workforce stability in hospitals and community health settings. These outcomes are particularly important in Mongolia, where shortages of healthcare personnel and limited training resources challenge the delivery of quality care. Enhancing readiness for practice through curriculum reform and strengthened clinical partnerships may therefore result in long-term cost savings and improve the efficiency of the healthcare system, offering important considerations for policymakers and institutional leaders.

## 5. Limitations

While this study contributes valuable insights into the factors influencing readiness for practice among baccalaureate nursing students in Mongolia, several limitations should be acknowledged. First, the study’s scope focused on baccalaureate nursing students only so the result should be generalized with caution. Second, the study may be limited by social desirability in all self-reported measures, which may introduce response bias. Third, because the quantitative data were collected via an online survey, there is a potential for selection bias. Participants with better internet access or higher motivation to participate may differ systematically from non-respondents, which could affect the representativeness of the sample. Fourth, the study did not account for external factors such as changes in healthcare policies or educational curricula that may have occurred during the research period, potentially impacting the results. The majority of participants in this study were female (97.3%), and gender was not a significant predictor of nursing students’ readiness for practice (β = −0.02, *p* = 0.702), reflecting the predominance of women in the Mongolian nursing workforce. This gender imbalance limits the generalizability of findings related to gender and may reduce the validity of regression estimates involving this variable. Future research should strive for a more balanced gender representation to more accurately assess potential gender differences in readiness for practice.

## 6. Implications for Nursing Education

The findings of this study have important implications for nursing education. Nursing programs should enhance curricula to strengthen students’ clinical competence, critical thinking, and ethical and cultural sensitivity. Incorporating learning activities that foster professional values, adaptability, and coping skills may further support readiness for practice. Additionally, collaboration between academic institutions and healthcare facilities is essential to provide students with supportive clinical learning environments that facilitate a smooth transition from student to professional nurse.

## 7. Conclusions

This study illuminates the intricate interplay of factors influencing readiness for practice among baccalaureate nursing students in Mongolia. The integration of quantitative and qualitative results enhances the depth of understanding, emphasizing the complementary nature of these research approaches. While recognizing the study’s limitations, the findings provide a foundation for targeted interventions and improvements in nursing education, ultimately contributing to the preparation of competent and resilient nursing professionals.

## Figures and Tables

**Table 1:** Sample characteristics (*N* = 150).

Characteristics	Categories	*n* (%)	Mean ± SD	Range
Gender	Female	146 (97.3)		
	Male	4 (2.7)		
Age (year)			24.15 ± 4.56	19–42
Marital status	Single	65 (43.3)		
	Married	85 (56.7)		
Pursuing second degree	Yes	8 (5.3)		
	No	142 (94.7)		
Location of school	Capital area	98 (65.3)		
	Rural area	52 (34.7)		
School status	Public	79 (52.7)		
	Private	71 (47.3)		
Previous employed experiences	Yes	20 (13.3)		
	No	130 (86.7)		
Previous job experiences	Assistant nurse	6 (4.0)		
	Volunteer worker	1 (0.7)		
	Dentist assistant	1 (0.7)		
	Other	12 (8.0)		
Current employment	Yes	39 (26.0)		
	No	111 (74.0)		
Motivation to choose the nursing profession	Academic interest and aptitude	85 (56.6)		
	Suggestion of parents	33 (22.0)		
	Easy to get scholarship	10 (6.6)		
	Depending ES	13 (8.6)		
	Other	9(6.2)		
Current GPA (total)			3.12 ± 0.32	2.30–3.80

**Table 2:** Descriptive statistics of readiness for practice-associated factors (*N* = 150).

Variables	Possible Range	Actual Range	Mean ±SD
Readiness for practice (20 items, 4-point scale)	20–80	39–79	57.8 ± 5.8
Clinical learning environment (34 items, 5-point scale)	34–170	72–170	136.3 ± 20.7
Clinical nursing competence (45 items, 5-point scale)	45–225	105–225	174.2 ± 21.9
Critical thinking (27 items, 5-point scale)	27–135	68–135	95.9 ± 14.7
Professional value (26 items, 5-point scale)	26–130	78–130	106.8 ± 12.3

**Table 3:** Correlation between readiness for practice and its associated factors (*N* = 150).

Variables	1	2	3	4	5	6	7
				*r* (*p*)			
1. Age							
2. Enrollment score	−0.190 * (0.020)						
3. GPA	0.213 ** (0.009)	0.083 (0.315)					
4. Clinical learning environment	0.160 (0.050)	0.037 (0.650)	−0.143 (0.082)				
5. Clinical nursing competence	0.006 (0.094)	0.211 ** (0.009)	0.152 (0.064)	0.476 ** (0.000)			
6. Critical thinking	−0.161 * (0.049)	0.129 (0.116)	−0.001 (0.989)	0.272 ** (0.001)	0.494 ** (0.000)		
7. Professional value	−0.183 * (0.025)	0.109 (0.183)	−0.058 (0.482)	0.306 ** (0.000)	0.449 ** (0.000)	0.321 ** (0.000)	
8. Readiness for practice	0.094 (0.252)	0.027 (0.743)	0.016 (0.848)	0.424 ** (0.000)	0.483 ** (0.000)	0.449 ** (0.000)	0.188 * (0.021)

Note: ** *p* < 0.01, * *p* < 0.05.

**Table 4:** Regression analysis of factors affecting nursing students’ readiness for practice (*N* = 150).

Variable	β	b	SE	t	*p*
Previous employed experience	0.10	0.00	0.06	0.12	0.898
Current employment	0.14	0.09	0.05	1.74	0.083
Gender	−0.02	−0.04	0.12	−0.38	0.702
Marital status	−0.16	−0.09	0.04	−2.10	0.057
Reason to choose the profession	−0.09	−0.01	0.01	−1.40	0.161
School of status	0.13	0.08	0.05	1.59	0.112
School of location	−0.03	0.01	0.05	−0.35	0.721
Financial support	0.01	0.00	0.02	0.14	0.885
Clinical learning environment	0.24	0.11	0.03	3.28	0.001
Clinical competence	0.17	0.86	0.04	1.95	0.005
Critical thinking	0.28	0.13	0.03	3.72	0.000
Professional value	−0.04	−0.02	0.04	−0.61	0.537
F = 8.92 (*p* < 0.001); R^2^ = 0.460; adjusted R^2^ = 0.409; VIF = 1.09–1.97.					

Note. β = Standardized regression coefficient; b = Unstandardized regression coefficient; SE = Standard error; VIF = Variance inflation factor.

**Table 5:** Integration of FGI participants’ perceptions in terms of nursing students’ readiness for practice.

Categories	Subcategories	Illustrative Quotations
Maturity	Coping ability to challenging clinical experiences	“Students who stay calm and think critically under pressure handle patients more safely.”
	Adaptability to the workplace	“Able to adjust quickly to changing ward conditions.”
	Genuinely desiring to learn	“Motivated students actively seek feedback and demonstrate professional growth.”
Competence	Essential clinical proficiency	“Students who demonstrate accurate clinical skills gain confidence in practice.”
	Ethical and cultural competence	“Awareness of cultural norms and ethical considerations is essential for patient care.”
	Communication competence	“Effective communication helps students collaborate within healthcare teams.”
Theory–practice integration	Theory application to nursing practice	“Applying classroom knowledge to real patient care shows readiness.”
	Practicing total aspects of care	“Students who consider all aspects of care provide better patient outcomes.”
	Adjust to changing patient situations	“Quick adaptation to unexpected patient changes is key.”
Professionalism	Passion for nursing	“Students who are passionate about nursing naturally engage more in patient care.”
	Understanding the value of nursing	“Recognizing the role of nursing in society demonstrates professional maturity.”

**Table 6:** Joint display with merged results of factors affecting nursing students’ readiness for practice.

Quantitative Findings (*n* = 150).	Qualitative Findings (Nurses *n* = 15, Faculty *n* = 10)	Mixed Method Interpretation
Clinical learning environment (β = 0.24, *p* = 0.001)	- Supportive clinical environments - Guidance from preceptors - Opportunities for hands-on practice	Expansion: - Qualitative findings expand on the quantitative results by providing contextual details on how the clinical environment supports readiness for practice
Clinical competence (β = 0.17, *p* = 0.005)	Competence - Essential clinical proficiency - Ethical and cultural competence - Communication competence in challenging environments	Confirmation & Expansion: - Quantitative findings of clinical competence were confirmed by qualitative data. - Expanded by highlighting ethical, cultural, and communication aspects as critical components
Critical thinking (β = 0.28, *p* = 0.000)	- Theory-practice integration - Applying nursing knowledge to patient care - Adjusting to changing patient conditions	Confirmation & Expansion: - Quantitative critical thinking factor confirmed by qualitative findings. - Expanded by emphasizing the application of knowledge in diverse clinical situations
Professional value (β = −0.04, *p* = 0.537)	- Passion for nursing - Understanding the value of nursing - Maturity: coping with challenges, adaptability, desire to learn	Discordance & Interpretation: - Quantitative findings suggest professional value was not significant, whereas qualitative data highlighted its importance. - This discordance indicates that professional value may not yet be reflected in measurable behaviors but is perceived as crucial by participants. - Expanded by qualitative insights on maturity, coping, and adaptability

## Data Availability

All data are included within the article, and additional data are available from the corresponding author upon reasonable request.

## Public Involvement Statement

There was no public involvement in any aspect of this research.

## Guidelines and Standards Statement

This manuscript followed the Mixed Methods Article Reporting Standards (MMARS).
